# LC–Q–TOF–MS/MS Identification of Specific Non-Meat Proteins and Peptides in Beef Burgers

**DOI:** 10.3390/molecules24010018

**Published:** 2018-12-21

**Authors:** Beata Mikołajczak, Emilia Fornal, Magdalena Montowska

**Affiliations:** 1 Department of Meat Technology, Poznan University of Life Sciences, Wojska Polskiego 31, Poznań, 60-624, Poland; magdalena.montowska@up.poznan.pl; 2 Department of Pathophysiology, Medical University of Lublin, Jaczewskiego 8b, Lublin, 20-090, Poland; emilia.fornal@umlub.pl

**Keywords:** food authenticity, beef burgers, soy, pea, milk and beetroot proteins, allergenic proteins, peptide markers, LC-Q-TOF-MS/MS

## Abstract

Beef burgers are a popular food choice, due to their taste and convenience. The extensive range of beef burgers with different flavours currently offered on the market is adding to their growing consumption. This study detected and identified specific non-meat proteins and peptide markers originating from functional preparations, i.e., powdered mixes of protein additives and spices, used as meat substitutes in the production of ready-to-cook beef burgers. Twenty-eight soy proteins, including isoforms (nine milk-, three pea- and one beetroot-specific protein) were found concurrently with a set of peptide markers unique to soy glycinin and β-conglycinin, pea vicilin and provicilin, milk α_S1_-casein, β-lactoglobulin, as well as beetroot elongation factor 2. Soy and beetroot proteins and peptides were observed in all burgers containing additives. Milk and pea proteins were included in powdered mixes but were not detected in burgers, indicating that their content was below the limit of detection. The study demonstrates that the proposed method can be implemented to analyse protein additives in cooked burgers; however, the presence of low amounts of additives, below 1–2%, should be further confirmed by using a more sensitive triple quadrupole instrument.

## 1. Introduction

Food products of animal origin constitute one of the most important segments of the food economy worldwide. Global bovine production and its consumption are in third place, after poultry and pork, and have shown a continuing upward trend since 1970. Projected data show that beef consumption will increase by 1.8% in 2015–2030 [1]. Globalisation and the consequent increased exposure to Western culture, organisational changes, flexible working hours and the increasing presence of women in the workforce have influenced the shift in many consumer preferences and resulted in expanding consumer interest in convenience food. Beef burgers are among the most popular products in the convenience food market, owing to the taste and ease of preparation. The use of ready-to-cook or ready-to-eat burgers minimises the time and effort of the consumer required for meal preparation [2]. A survey conducted by Shan et al. [3] revealed that 20.2% of 481 consumers from the Republic of Ireland consumed beef burgers once a week while for 40.5% of the cohort, the frequency was 1–3 times per month.

Currently, there is a great demand for healthy meat products, that are, on the one hand, low in fat, cholesterol, salt, nitrites and energy content, but on the other hand, are enriched in health-promoting bioactive compounds, such as unsaturated fatty acids, sterols, bioactive peptides, flavonoids and fibre [4]. Therefore, manufacturers are exploring new functional preparations to develop high-quality products with low sodium content (below 1.2%) as well as different sensory attributes, flavour and texture, according to consumer demands. Currently, burgers can be produced with interesting combinations of additives that besides reducing the costs, also enhance flavour and improve the quality characteristics. The addition of non-meat proteins (i.e., soy, milk, pea, egg white), aromatic herbs and spices, and extracts from plants and fruits, is becoming increasingly popular in beef burger production. These additives have different functional roles in the food matrix.

Legume proteins (soy, pea, bean) are commonly used in meat products to increase water holding capacity and improve the sensorial characteristics, such as texture (firmness, juiciness, sliceability). Soy proteins are also incorporated both as meat and animal fat substitutes for health reasons. These ingredients also serve an economic purpose—reducing the production cost while maintaining the high protein content in the product [5,6]. The addition of up to 3–5% soy protein increases the cooking yield and the weight of the final product [6,7].

All types of milk proteins, i.e., caseins, caseinates and whey proteins, and mixtures thereof, act as good water and fat binders and stabilisers. Various casein or whey ingredients are usually declared as “milk proteins” on the product label [8]. Previous work showed that milk proteins decreased the amount of free water and increased the immobilised water in comminuted frankfurter batters, thereby increasing the shear force value and firmness, and final textural modification of the frankfurters [9]. Among the milk proteins, β-casein is the most effective stabiliser because it reduces the surface tension to the greatest extent [10]. Moreover, enzymatic hydrolysates of soy or milk proteins contain various physiologically functional peptides [11]. For instance, the addition of 20 mg/mL casein peptides, obtained by the proteolytic enzymes Alcalase^®^ and Flavourzyme^®^, to ground beef homogenates, effectively inhibited lipid oxidation [12].

Various vegetable powders and extracts that are rich in natural antioxidants (i.e., flavonoids, phenolic compounds) enhance the healthful properties and quality of meat products [13]. For example, vegetable powders obtained from spinach, yellow pea, onion, red pepper, green pea and tomato improved the oxidative stability of cooked turkey meat patties by 20% to 30% [14]. Flavonoid extracts from beetroot proved to be effective in reducing lipid oxidation in cooked pork patties [15]. Beetroot extract is the main source of betalains—water-soluble pigments—primarily used as food colourants. Besides, betalains have strong antioxidative properties and may also exert other important activities, such as anticarcinogenic and antiglycation [16].

Substitution of ingredients is the most frequent food adulteration and, consequently, less valuable ingredients of non-meat origin can be illegally added to meat burgers, to replace the most expensive components. These ingredients may also be the source of undeclared allergens responsible for severe allergic or food intolerance responses in allergic or sensitive consumers. Since modern meat products consist of an increasing number of non-meat components, there is a need for novel, reliable and sensitive analytical methods, to ensure the accuracy of labelling, reveal dishonest producers and for consumer safety. Most commonly applied immunochemical- and DNA-based methods have some limitations, especially when analysing multi-component and thermally processed food matrices, while modern instrumental mass spectrometry (MS)-based methods, which are characterised by high resolving power, sensitivity and specificity, have succeeded in the analysis of complex and processed meat products [17].

In this paper, MS-based proteomic and peptidomic analyses are presented to detect and identify non-meat proteins and specific peptide markers originating from functional preparations used in the production of beef burgers. Ready-to-cook commercially produced burgers containing different meat content, non-meat proteins and other additives—namely, soy, pea and milk proteins—and powdered onion and beetroot juices were examined. Proteins and peptides specific to the observed additives were identified by liquid chromatography–quadrupole–time-of-flight–tandem mass spectrometry (LC–Q–TOF–MS/MS).

## 2. Results and Discussion

### 2.1. Identification of Specific Non-Meat Proteins

Four types of industrially prepared, frozen, ready-to-cook beef burgers (samples B1–B4) were investigated in this study. The detailed composition of each product declared on the packaging label is shown in Table 1. It is well known that thermal treatment affects MS-based protein and peptide identification, due to protein denaturation, reduced protein solubility and aggregation processes. Therefore, in this work, the final identification and confirmation of the results were performed on cooked burgers (samples B5–B8), after storage at −18 °C for 1 month, for the detection of unmodified, thermally-resistant proteins and peptides. The studied burgers were manufactured from beef incorporated with the addition of three different powdered mixes of functional additives and spices, which included, among others, soy, milk and pea proteins, powdered onion and beetroot juices, wheat fibre, plant flavours and spices, as shown in Table 1. These three mixes were also analysed and used as reference samples M1–M3.

Protein digests were analysed using LC–Q–TOF–MS/MS and the Spectrum Mill searching algorithm. More than 110 animal proteins, as well as 33–40 vegetable proteins, were identified, depending on the analysed samples. Burgers B4 (raw) and B8 (cooked) were made from 100% meat. These burgers were used as the control groups for raw and cooked samples, respectively since they did not contain any functional preparations, as shown in Table 1. In general, the same meat and vegetable proteins were revealed in raw and cooked burgers, but protein sequence coverage was lower in the cooked samples. Table 2 provides a comparison of the Spectrum Mill identification results for soy-specific proteins between powdered mixes of protein additives and spices (M1–M3) and the corresponding cooked burgers. There was a predominance of 20 soybean proteins, including glycinin (G1, G2, G3 and G4 isoforms) and β-conglycinin (α, α’ and β chains) in all the examined samples, except for the control B4 and B8 burgers, characterised by a high (>45.5%) sequence coverage, as shown in Table 2. The same 20 soy proteins also occurred in raw burgers B1–B3. Soy proteins were partly substituted for meat in the burgers, which accounted for the large quantities of soy proteins present in the products.

Eight comparatively less abundant soy proteins, as well as pea-, milk- and beetroot-specific proteins found in powdered mixes of additives are presented in Table S1 (Supplementary Materials). Three pea-specific proteins, namely provicilin, vicilin and legumin, appeared only in mix M3. Nine milk proteins, i.e., β-lactoglobulin, α_S1_-casein, β-casein, lactotransferrin, butyrophilin, α-lactalbumin, lactadherin, κ-casein and milk glycoprotein PP3, were observed in mix M2 but two whey proteins (β-lactoglobulin and α-lactalbumin) were detected in mix M3. These proteins were identified with good confidence scores, showing a sequence coverage in the range of 5.3–81.4 (milk proteins) and 3–13 (whey proteins) matched peptides. The results are consistent with the mixes’ labels since the soy, milk or pea proteins were included in the list of ingredients for a given product. Possible traces of milk were declared on the M3 packaging. In addition, no onion proteins were found in the examined burgers and mixes (added to B1 and B5 at 1.78%). Moreover, onion is rich in simple sugars while low in protein. The lack of pea, milk and onion protein content in the ready-to-cook burgers was most likely because these ingredients were present in quantities below the limit of detection of the method.

Powdered beetroot juice was declared in all three mixes of additives and, consequently, several beetroot proteins were found in all types of ready-to-cook beef burgers (B1–B3) manufactured with given mixes and their cooked counterparts (B5–B7). However, only one protein, namely, elongation factor 2 (EF-2), turned out to be unique to beetroot when its sequence was searched against all entries in the NCBI (National Centre for Biotechnology Information) protein sequence database for species specificity. Eukaryotic EF-2 is a protein with a calculated molecular weight of 94,767 Da and 843 amino acid residues. It catalyses the guanosine triphosphatase-dependent ribosomal translocation step during translation elongation. In our mixes, the protein was identified with good confidence scores, 2–7 matched peptides and 3.9–10.9% protein sequence coverage. Total ion chromatograms of the three mixes of additives and spices are shown in Figure S1 (Supplementary Materials), and total ion chromatograms of the cooked burgers manufactured with the addition of their respective mixes are shown in Figure S2 (Supplementary Materials).

Some of the major food allergens, such as glycinin (Gly m 6) and β-conglycinin (Gly m 5) soy proteins, and α_S1_-casein (Bos d 8) and β-lactoglobulin (Bos d 5) milk proteins were detected within the study. These proteins have been determined previously in various commercial meat products, for example, frankfurters, chicken and pork liver sausage, and smoked and cooked chicken breast, by nano-LC–Q–TOF–MS/MS [17]. LC–QQQ–MS/MS in multiple reaction monitoring mode has been developed to detect soy (glycinin) and milk (α_S1_-casein, α_S2_-casein) allergens in wheat bread spiked with soy flakes and skim milk powder [18]. Hoffmann et al. [19] noticed soy glycinin, and pea convicilin, vicilin and provicilin proteins in emulsion-type pork sausages, using a recently developed LC–MS/MS method. Allergies to pea proteins are less frequent than allergies to soy and mainly refer to the proteins vicilin and convicilin [20]. Vicilin and provicilin storage seed proteins were found in our mix M3 of functional additives and spices. Considering that neither pea nor milk proteins were detected in raw and cooked burgers, using our untargeted LC–Q–TOF–MS/MS method confirms that the results may be affected mostly by food complexity, the limit of detection of the chosen method and industrial processing [21]. It is recommended that a more sensitive and selective triple quadrupole mass spectrometer should further be implemented to analyse less abundant protein components in beef burgers.

### 2.2. Identification of Specific Peptide Markers

The most intense, potentially unique, peptide markers of soy, cow milk, pea and beetroot occurring in the LC–Q–TOF–MS/MS profiles were searched for species and protein specificity, against all protein sequence records stored in the NCBI database, using the BLASTP algorithm. As a result, 47 unique peptides were most abundant, originating from soy glycinin G1, G2 and G3 isoforms and β-conglycinin, in all mixes, and raw and cooked beef burgers, as shown in Table 3. The Spectrum Mill protein score was over 449, and the peptide intensity ranged from 10^6^–10^8^. Figure S3 (Supplementary Materials) shows the mass spectrum of the peptide VFDGELQEGR (*m*/*z* 575.28, 2+) unique to soy glycinin G1 and obtained from a cooked burger (sample B5). For control groups of raw and cooked burgers (B4 and B8), no soy-, milk- and pea-specific peptides were discerned. These negative results confirm the correctness of the labelling.

**Figure 1:**
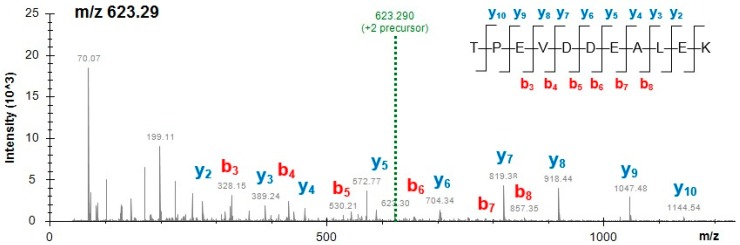
Mass spectrum of the milk β-lactoglobulin marker peptide TPEVDDEALEK (2+) obtained from functional additive preparation (mix M2).

Table 4 presents the peptides unique to cow milk, pea and beetroot obtained from powdered mixes of additives and spices. The peptides belong to some main food allergens, such as milk β-lactoglobulin, α-lactalbumin and α_S1_-casein while two unique peptides derived from pea allergenic proteins vicilin and provicilin. For example, the MS/MS spectrum of the milk β-lactoglobulin marker peptide TPEVDDEALEK (*m*/*z* 623.29, 2+) obtained from mix M2 is presented in Figure 1. Considering the large number of specific peptides detected in this study, and the limited space available in this article, some of the peptides are presented in the Supplementary Materials. Peptides specific to both cow milk and milk of other mammals are shown in Table S2 (Supplementary Materials), and the soy-specific peptides obtained from less abundant proteins and detected in all mixes and burgers are shown in Table S3 (Supplementary Materials). Figure S4 (Supplementary Materials) presents the mass spectrum of the pea provicilin-specific peptide VLLEQQEQEPQHR (*m*/*z* 545.28, 3+) obtained from mix M3.

**Figure 2:**
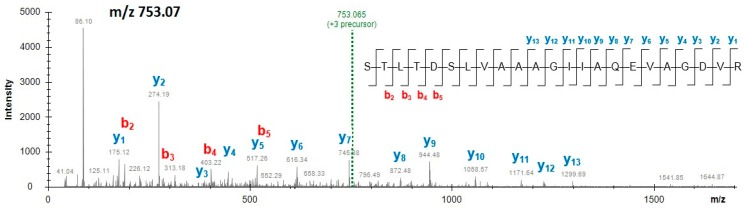
Mass spectrum of the beetroot elongation factor 2-specific peptide STLTDSLVAAAGIIAQEVAGDVR (3+) obtained from functional additive preparation (mix M1).

Among four unique peptides originating from beetroot EF-2 protein, as shown in Table 4, only one peptide STLTDSLVAAAGIIAQEVAGDVR *(m*/*z* 753.07, 3+) was detected in all samples of mixes and burgers, as shown in Figure 2. The other three peptides were identified in powdered mixes. Figures S5 and S6 (Supplementary Materials) display the mass spectra of the peptides unique to beetroot EF-2, that is, EGALAEENMR (*m*/*z* 560.25, 2+) and FFAFGR (*m*/*z* 372.69, 2+), respectively, acquired from mix M1. Powdered beetroot juice was added to the examined burgers containing a reduced meat content, to enhance the redness in products that incorporated a significant amount of meat replacers (i.e., soy, pea and milk protein additives, Table 1). Beetroot extracts are an increasingly popular component in the meat industry not only for their colour but also because of their antioxidant activity and positive impact on the preservation of the final meat product [14,15,22].

Unique peptides derived from non-meat proteins may be implemented for the simultaneous detection of soy, pea, milk and beetroot in complex food matrices. Previously, the same two soy peptides FYLAGNQEQEFLK (glycinin G1/G2) and SQSDNFEYVSFK (glycinin G1/G2/G3) found in this work, have been used to detect soy alongside six other allergenic commodities (milk, egg, hazelnut, peanut, walnut and almond) in bread [18], as well as soy, alongside egg, milk, hazelnut and peanut, in spiked cookie samples [23]. Peptide markers FYLAGNQEQEFLK (glycinin G1/G2), EAFGVNMQIVR (glycinin G2) and HFLAQSFNTNEDIAEK (glycinin G4) have been identified in various types of poultry meat products manufactured with the addition of functional protein preparations [17]. The same three soy peptides have been chosen for soy detection in emulsion-type sausages spiked with soy protein isolate [19]. Recently, the simultaneous quantification of meat and allergenic protein additives, including soy and milk, was conducted in a wide range of meat products (raw, cooked, smoked, sterilised), based on two glycinin marker peptides VLIVPQNFVVAAR (G1) and ISTLNSLTLPALR (G4), as well as bovine α_S1_-casein peptides YLGYLEQLLR and FFVAPFPEVFGK [24]. Moreover, some of the whey peptides presented herein, have been identified in meat products, cookies and dairy products [17,23,25,26]. The results of the present study confirm the applicability of MS-based methods to detect multiple components and allergens in beef burgers.

Mass spectrometry is a robust technique that can differentiate between accidental contamination that may be a source of hidden allergens and intentional addition to meat products prepared with non-meat ingredients used for the replacement of the most expensive components of beef burgers, and thus is a reliable technique to detect meat adulterations [17,18,19,24]. However, to increase the sensitivity of the analysis, the method should be transferred to a more sensitive QQQ mass spectrometer and validated in multiple reaction monitoring mode.

## 3. Materials and Methods

### 3.1. Preparation of Samples

Four types of commercially-available, ready-to-cook, frozen beef burgers were manufactured in a local meat processing plant in the Wielkopolska region, Poland. Each packet contained 10 eighty-gram burgers of 1 cm thickness. After purchase, the frozen burgers were coded, vacuum-packed in polyethylene bags and kept at −18 °C for future peptidomic analyses. Three burgers from each type of beef burgers were analysed. Each type differed mainly in the content of meat, protein additives and spices. Table 1 lists the detailed composition of the examined burgers presented on the product label. Minced beef used for the manufacture of burgers met the requirements of the European Union Regulation 1169/2011 [8] concerning the content of fat (≤20%) and the collagen/meat proteins ratio (≤15%). The beef content in the control group B4 was 100%. Proteins of five species, namely, soy (*Glycine max*), cow milk (*Bos taurus*), pea (*Pisum sativum*), onion (*Allium cepa*) and beetroot (*Beta vulgaris*) were examined in the present study.

All types of burger samples were heated in a convection-steam oven (model SCC 61E, Rational International AG, Heerbrugg, Germany) at 180 °C for 9 min. Raw (samples B1–B4) and cooked beef burgers (B5–B8) were analysed in triplicate. Additionally, three powdered mixes of functional preparations, consisting of various protein additives and spices (samples M1–M3) that were used to produce the beef burgers, as shown in Table 1, were analysed as reference samples.

### 3.2. Protein Digestion

Samples (0.3 g) were homogenised in 0.1 mol/L of aqueous ammonium bicarbonate, using a T25 Ultra-Turrax (IKA Labortechnik, Staufen, Germany) at 9500 rpm for 2 × 20 s and then vacuum-dried using a miVac Duo Concentrator (Genevac Ltd., Ipswich, UK). Dried samples of functional preparations (5 mg) and burgers (10 mg) were rehydrated in 100 µL of 0.1 mol/L ammonium bicarbonate (1 h). The proteins were reduced by 0.2 mol/L dithiothreitol (DTT) at 56 °C for 1 h and then alkylated using iodoacetamide (IAA), at room temperature for 30 min in the dark. The remaining IAA was quenched by the addition of 0.2 mol/L DTT and incubation at room temperature for 30 min. The samples were digested in an ammonium bicarbonate solution (pH 8.3) containing 0.083 µg/µL trypsin, at 37 °C, overnight (18 h). The digests were purified by reversed-phase extraction, using Sep-Pak C18 Plus cartridges (Waters, Milford, MA, USA). The dried (miVac Duo concentrator) eluates were resuspended in 0.1% formic acid in milli-Q water (solvent A), before analysis by LC–Q–TOF–MS/MS.

### 3.3. LC–Q–TOF–MS/MS Analysis

The ultra-high-performance LC–Q–TOF–MS/MS analysis was carried out using an Agilent Technologies (Santa Clara, CA, USA) 1290 Infinity Series liquid chromatograph coupled with an Agilent Technologies 6550 iFunnel Q–TOF LC/MS device equipped with an electrospray ionisation Agilent Technologies Jet Stream ion source. Chromatographic separation was achieved on an Agilent Zorbax Eclipse Plus C18 RRHD column (2.1 × 150 mm, 1.8 µm pore size) (Agilent, Santa Clara, CA, USA). The injection volume was 10 µL. The mobile phase consisted of 0.1% formic acid in milli-Q water (solvent A) and 0.1% formic acid in 98% acetonitrile/milli-Q water (solvent B) at a flow rate of 0.3 mL/min. The mobile phase gradient (3–97% B) steps were applied as follows: 0–1 min, 3% B; 1–40 min, 5% B; 40–45 min, 40% B; 45–55 min, 90% B, and a 5 min post-run at 3% B. The source nitrogen gas temperature was 250 °C, the sheath gas flow was 11 L/min, and the nebuliser pressure was 35 psig. The voltages were set at 3500 (capillary), 1000 (nozzle) and 400 V (fragmentor). Positive ions were acquired in the range of 100–1700 *m/z* for MS scans, and 40–1700 *m/z* for auto MS/MS scans, at a scan rate of 5 scans/s for MS and 3 scans/s for MS/MS, respectively. Internal mass correction was enabled, by using two reference masses at 121.0509 and 922.0098 *m/z*. Instrument control and data acquisition were performed using Agilent MassHunter Workstation software.

### 3.4. Protein and Peptide Identification

A UniProtKB/Swiss-Prot database search for protein and peptide identification was performed, using the Spectrum Mill MS Proteomics Workbench, using a 20 ppm precursor mass tolerance, 50 ppm product mass tolerance and >70% precursor isolation purity. Search parameters were set as follows: trypsin enzyme, taxonomy all entries, one missed cleavage, carbamidomethylation as fixed modification, methionine oxidation as a variable modification and >70% score peak intensity. The matches and Spectrum Mill scores were evaluated at 1% of the false discovery rate (FDR), for identity and homology threshold. Protein identification corresponding to protein sequences with a minimum of two unique peptides was accepted [27,28]. Selected peptides in FASTA format (amino acid residues are represented using single-letter codes) were searched against the NCBI non-redundant protein database using the protein BLAST alignment research tool and BLASTP algorithm (USA National Library of Medicine, Bethesda, MD, USA), for species and protein specificity [29].

## 4. Conclusions

Beef burgers—convenience food products with a high frequency of consumption—should be of good quality, which relies on close monitoring by adequate food agencies and regulatory bodies. The escalating inclusion of non-meat ingredients in meat products, on the one hand, contributes to increasing the range of products on the market and the market size, and on the other hand, can add to the growing number of cases of food intolerance. In this study, an LC–Q–TOF–MS/MS method for multi-component detection of soy, pea, milk and beetroot in ready-to-cook and cooked beef burgers supplemented with functional preparations was examined. Specific soy, pea, milk and beetroot proteins were detected, including a set of unique peptides originating from allergenic proteins, namely, soy glycinin and β-conglycinin, pea vicilin and provicilin, and milk α_S1_-casein and β-lactoglobulin. These peptides can be used as peptide markers of food authenticity. The method can be implemented to study complex and processed food matrices, and thus can be an alternative approach to various enzyme-linked immunosorbent assays and polymerase chain reaction tests.

## Figures and Tables

**Table 1:** The composition of beef burgers declared on the packaging.

Ingredient (%)	Burger B1/B5	Burger B2/B6	Burger B3/B7	Burger B4/B8
Beef	51	51	80	100
Fat	≤20	≤20	≤20	≤20
Collagen	≤25	≤25	≤25	≤15
Proteins	11.5	13.1	16.0	19.0
NaCl	0.95	1.1	0.8	n.d.^1^
**Mix**	**M1**	**M2**	**M3**	
**Non-meat proteins**	Soy proteins rehydrated (30%)	Soy proteins rehydrated (24%) Milk proteins	Soy proteins Pea proteins Traces of milk proteins	n.d.
**Other non-meat additives**	Powdered onion 1.78%, water, sodium chloride, wheat fibre, plant flavours, breadcrumbs, monosodium glutamate, powdered beetroot juice, spices	Water, sodium chloride, wheat fibre, plant flavours, breadcrumbs, powdered beetroot juice, spices	Water, sodium chloride, plant flavours, powdered beetroot juice, dextrose	n.d.

^1^ n.d.: not declared.

**Table 2:** Identification results for soy proteins from powdered mixes of protein additives (M1–M3) and corresponding cooked burgers (B5–B7).

Identified Protein	Accession No.	Mass (Da)	Matched Peptides ^1^			Sequence Coverage (%) ^2^			Matched Peptides			Sequence Coverage (%)		
			M1	M2	M3	M1	M2	M3	B5	B6	B7	B5	B6	B7
Seed lipoxygenase-1	P08170	94,595.8	46	38	44	74.2	63.1	68.6	19	23	7	36.4	43.7	11.6
Seed lipoxygenase-2	P09439	97,429.1	47	34	45	73.8	52.7	71.5	18	5	7	28.5	5.9	9.3
Seed lipoxygenase-3	P09186	97,155.8	53	40	46	61.9	60	64	22	27	15	41.8	48.8	26.7
β-Conglycinin, α chain	P13916	70,577.1	39	32	36	58.8	57	57	28	28	25	54.4	53.3	51.5
β-Conglycinin, α′ chain	P11827	74,609.2	27	20	29	44.7	37.8	48.6	19	15	16	33.9	28.4	29.5
β-Conglycinin, β chain	P25974	50,608.1	29	27	28	68.1	66.9	68.1	18	21	14	59.9	61.2	48.9
Glycinin	P04347	54,960.3	20	18	19	62	55.8	62	16	16	25	52.3	52.3	51.5
Glycinin G1	P04776	54,868.3	29	28	28	83	83	81.4	24	24	23	80.2	79.3	75.9
Glycinin G2	P04405	64,042.4	28	26	27	83.7	83.7	75.4	20	22	20	73.6	82.6	71.5
Glycinin G3	P11828	58,411.5	24	21	26	80	73.5	80	17	17	11	71.5	64.2	45.5
Glycinin G4	P02858	56,332.9	31	28	29	61.3	65.3	61.3	21	23	20	53.0	53.2	52.8
Sucrose-binding protein	Q04672	60,920.9	24	18	26	56.1	45	58.9	16	18	13	41.4	47.5	29.3
Trypsin inhibitor A	P01070	24,290.1	14	15	15	56.4	60.1	58.7	11	11	11	53.7	49	49.5
Kunitz-type trypsin inhibitor KTI1	P25272	22,830.8	9	8	11	47.7	36.9	54.1	5	4	4	34.4	23.6	23.6
β-Amylase	P10538	56,484.1	14	10	14	39.1	30	35	3	3	1	10.2	10.2	5
Lectin	P05046	30,927.4	10	11	10	71.9	72.9	71.9	8	8	5	60.7	64.2	35
2S albumin	P19594	19,030.2	8	7	7	41.7	41.7	41.7	6	6	5	41.1	41.1	32.9
P24 oleosin isoform B	P29531	23,392.2	7	7	7	30.4	30.4	30.4	4	3	3	18.8	13	13
P34 probable thiol protease	P22895	43,135.4	6	6	6	22.1	22.1	22.1	5	5	4	21.8	21.8	21.6
Basic 7S globulin	P13917	47,133.9	14	13	14	57.1	54.5	57.1	12	13	7	50.1	51.7	29.9

^1^ Number of matched peptides in the database search; ^2^ Percentage of coverage of the protein amino acid sequence.

**Table 3:** Peptides unique to glycinin G1, G2 and G3 isoforms, and β-conglycinin detected in all analysed samples of mixes and burgers.

Parent Ion (*m*/*z*)	Mr (exp)	Exp z ^1^	Peptide Score ^2^	Total Intensity Range	Peptide Marker	Protein	Protein Score ^3^
686.8540	1372.7002	2	19.99	1.23 × 10^6^–1.01 × 10^8^	(R)ALIQVVNCNGER(V)		558.84
765.3553	1529.7013	2	18.57	3.05 × 10^6^–7.95 × 10^7^	(R)EQPQQNECQIQK(L)		
586.8197	1172.6310	2	20.15	1.87 × 10^7^–5.76 × 10^8^	(K)FLVPPQESQK(R)	Glycinin G1	
889.7510	2667.2330	3	22.57	1.84 × 10^7^–8.57 × 10^8^	(K)GIFGMIYPGCPSTFEEPQQPQQR(G)		
820.1402	3277.5284	4	22.42	3.19 × 10^7^–9.40 × 10^8^	(K)HQQEEENEGGSILSGFTLEFLEHAFSVDK(Q)		
554.3123	1107.6157	2	19.15	2.14 × 10^5^–1.10 × 10^7^	(R)LSAEFGSLR(N)		
616.7735	1232.5390	2	16.95	1.22 × 10^6^–3.56 × 10^7^	(K)NLQGENEGEDK(G)		
1141.5955	3422.7624	3	22.17	1.76 × 10^7^–1.60 × 10^9^	(K)TNDTPMIGTLAGANSLLNALPEEVIQHTFNLK(S)		
575.2810	1149.5535	2	21.65	3.21 × 10^6^–2.30 × 10^8^	(R)VFDGELQEGR(V)		
713.4353	1425.8576	2	24.28	2.64 × 10^7^–4.45 × 10^8^	(R)VLIVPQNFVVAAR(S)		
709.3268	1417.6455	2	19.51	1.15 × 10^5^–1.90 × 10^7^	(K)YQQEQGGHQSQK(G)		
793.8984	1586.7849	2	23.48	1.53 × 10^7^–3.68 × 10^8^	(R)FYLAGNQEQEFLK(Y)	Glycinin G1/G2	558.84/549.21
725.8287	1450.6485	2	21.39	1.94 × 10^7^–3.82 × 10^8^	(R)SQSDNFEYVSFK(T)	G1–G3	558/549/449
1246.6297	2492.2503	2	22.62	9.73 × 10^5^–1.61 × 10^8^	(K)NAMFVPHYNLNANSIIYALNGR(A)	Glycinin G1/G3	558.84/449.67
679.8461	1358.6845	2	19.95	6.93 × 10^5^–6.69 × 10^7^	(R)ALVQVVNCNGER(V)		
632.3305	1263.6514	2	19.95	1.33 × 10^6^–3.23 × 10^8^	(K)EAFGVNMQIVR(N)	Glycinin G2	549.21
897.7005	2691.0846	3	19.95	4.33 × 10^5^–1.16 × 10^8^	(R)KPQQEEDDDDEEEQPQCVETDK(G)		
497.7697	994.5316	2	18.63	4.55 × 10^6^–8.60 × 10^7^	(K)LSAQYGSLR(K)		
1240.1321	2479.2551	2	20.70	1.54 × 10^6^–1.56 × 10^8^	(K)NAMFVPHYTLNANSIIYALNGR(A)		
966.4660	1931.9193	2	24.50	1.12 × 10^7^–6.50 × 10^8^	(R)NLQGENEEEDSGAIVTVK(G)		
937.4649	1873.9191	2	20.23	1.11 × 10^7^–6.50 × 10^8^	(K)NNNPFSFLVPPQESQR(R)		
915.9747	3660.8544	3	19.69	1.03 × 10^7^–4.26 × 10^8^	(R)QNIGQNSSPDIYNPQAGSITTATSLDFPALWLLK(L)		
1183.5625	2366.1147	2	22.92	1.41 × 10^7^–5.19 × 10^8^	(K)QQEEENEGSNILSGFAPEFLK(E)		
1201.1432	2401.2762	2	22.88	3.55 × 10^7^–9.17 × 10^8^	(R)VFDGELQEGGVLIVPQNFAVAAK(S)	Glycinin G2	
747.8511	1494.6932	2	17.89	3.91 × 10^5^–3.49 × 10^7^	(K)YQQQQQGGSQSQK(G)		549.21
1116.0425	2231.0768	2	20.69	4.61 × 10^5^–7.80 × 10^7^	(R)FYLAGNQEQEFLQYQPQK(Q)	Glycinin G3	449.67
1205.6108	3614.8126	3	20.39	3.05 × 10^6^–2.46 × 10^8^	(R)HNIGQTSSPDIFNPQAGSITTATSLDFPALSWLK(L)		
1045.4962	3134.4701	3	24.92	8.48 × 10^5^–1.43 × 10^8^	(R)QQEEENEGGSILSGFAPEFLEHAFVVDR(Q)		
867.2119	3465.8085	4	19.47	1.11 × 10^6^–2.79 × 10^7^	(K)TNDRPSIGNLAGANSLLNALPEEVIQQTFNLR(R)		
1047.1164	2093.2216	2	22.67	3.90 × 10^7^–2.59 × 10^8^	(K)AIVILVINEGDANIELVGLK(K)	β-Conglycinin, α chain	675.52
901.1371	2701.3906	3	22.17	1.31 × 10^7^–3.61 × 10^8^	(R)DLDIFLSIVDMNEGALLLPHFNSK(A)		
1013.4783	2025.9472	2	23.37	3.79 × 10^6^–1.48 × 10^8^	(K)EQQQEQQQEEQPLEVR(K)		
592.2857	1183.5630	2	16.74	2.38 × 10^6^–1.38 × 10^8^	(R)ESYFVDAQPK(K)		
1124.9099	3372.7070	3	19.96	2.48 × 10^7^–1.45 × 10^9^	(R)NFLAGSQDNVISQIPSQVQELAFPGSAQAVEK(L)		
526.2697	1051.5320	2	14.05	2.03 × 10^6^–2.36 × 10^8^	(K)NPFLFGSNR(F)		
1076.5199	2152.0305	2	23.11	2.01 × 10^7^–5.28 × 10^8^	(R)VPSGTTYYVVNPDNNENLR(L)		
622.8594	1244.7096	2	20.04	1.31 × 10^7^–2.47 × 10^8^	(R)LQESVIVEISK(E)	β-Conglycinin, α/α’ chain	675.52/482.98
577.2909	1153.5735	2	18.62	5.51 × 10^6^–1.82 × 10^8^	(R)NILEASYDTK(F)		
698.4148	2093.2216	3	18.22	7.53 × 10^6^–4.52 × 10^7^	(K)AIVVLVINEGEANIELVGIK(E)	β-Conglycinin, α’ chain	482.98
903.1223	2707.3436	3	21.01	5.11 × 10^6^–3.15 × 10^8^	(R)DLDVFLSVVDMNEGALFLPHFNSK(A)		
669.8188	1338.6284	2	18.65	9.42 × 10^5^–1.41 × 10^7^	(R)DSYNLQSGDALR(V)		
1215.5866	2430.1612	2	21.58	4.20 × 10^5^–1.29 × 10^8^	(R)FESFFLSSTQAQQSYLQGFSK(N)		
1047.1164	2093.2216	2	22.67	3.90 × 10^7^–2.59 × 10^8^	(K)AIVILVINEGDANIELVGIK(E)	β-Conglycinin, β chain	546.79
886.4683	2657.3821	3	22.61	6.34 × 10^6^–2.00 × 10^8^	(R)DLDIFLSSVDINEGALLLPHFNSK(A)		
615.3408	1229.6736	2	16.74	1.54 × 10^6^–1.16 × 10^8^	(R)QQEGVIVELSK(E)		
887.4435	1773.8766	2	18.78	1.79 × 10^6^–1.12 × 10^8^	(R)QVQELAFPGSAQDVER(L)		
478.7618	956.5160	2	17.37	1.40 × 10^6^–5.29 × 10^7^	(R)SPQLENLR(D)		

^1^ Parent ion charge state; ^2^ Spectrum Mill peptide score at 1% false discovery rate (FDR); ^3^ Spectrum Mill protein score at 1% FDR.

**Table 4:** Peptides specific to cow milk, pea and beetroot obtained from mixes of additives.

Parent Ion (*m/z*)	Mr (exp)	Exp z	Peptide Score	Total Intensity Range	Peptide Marker	Protein	Protein Score
**Milk Peptides**							
858.4109	1715.8057	2	20.69	8.80 × 10^6^–6.91 × 10^8^	(R)LSFNPTQLEEQCHI(-)	β-Lactoglobulin	245.95
623.2994	1245.5845	2	22.62	4.95 × 10^7^–1.49 × 10^8^	(R)TPEVDDEALEK(F)		
772.7189	2316.1369	3	18.97	3.77 × 10^7^–4.27 × 10^7^	(K)EPMIGVNQELAYFYPELFR(Q)	α_S1_-Casein	106.74
692.8696	1384.7300	2	18.97	4.15 × 10^7^–4.61 × 10^7^	(R)FFVAPFPEVFGK(E)		
880.4773	1759.9450	2	20.53	4.35 × 10^7^–4.82 × 10^7^	(K)HQGLPQEVLNENLLR(F)		
374.2062	747.4036	2	10.70	3.30 × 10^5^–7.24 × 10^5^	(R)APVDAFK(E)	Lactotransferrin	56.56
638.8128	1276.6168	2	17.53	1.09 × 10^6^–1.19 × 10^6^	(R)NPDEEGLFTVR(A)	Butyrophilin subfamily 1 member A1	80.89
481.2771	961.5465	2	16.66	1.11 × 10^6^–2.21 × 10^6^	(K)VSPAVFVSR(E)		
854.9494	1708.8905	2	14.72	9.73 × 10^5^–2.10 × 10^6^	(K)INLFDTPLETQYVR(L)	Lactadherin	40.87
374.2060	1120.6010	3	14.82	4.33 × 10^5^–7.64 × 10^5^	(R)IQPVAWHNR(I)		
990.5502	1980.0913	2	16.02	6.20 × 10^6^–1.21 × 10^7^	(R)SPAQILQWQVLSNTVPAK(S)	κ-Casein	34.99
630.8260	1260.6430	2	14.40	3.86 × 10^6^–4.31 × 10^6^	(R)NLQISNEDLSK(E)	Glycosylation-dependent cell adhesion molecule 1	43.40
421.8969	1263.6732	3	16.35	1.23 × 10^7^–1.32 × 10^7^	(K)SLFSHAFEVVK(T)		
**Pea Peptides**							
545.2826	1633.8293	3	12.27	5.19 × 10^5^–9.63 × 10^5^	(K)VLLEQQEQEPQHR(R)	Provicilin (Fragment)	59.72
957.9525	1914.8967	2	12.61	1.06 × 10^6^–1.16 × 10^6^	(K)NILEASFNTDYEEIEK(V)	Vicilin	47.03
**Beetroot Peptides**							
753.0750	2257.2034	3	21.82	1.09 × 10^6^–9.85 × 10^6^	(K)STLTDSLVAAAGIIAQEVAGDVR(M)	Elongation factor 2 (EF-2)	88.19
974.1526	2920.4649	3	15.64	2.03 × 10^6^–2.11 × 10^6^	(K)VIENANVIMATYEDPLLGDVQVYPEK(G)		
560.2594	1119.5099	2	14.72	2.90 × 10^5^–7.04 × 10^5^	(K)EGALAEENMR(G)		
372.6955	744.3828	2	11.03	8.55 × 10^5^–1.69 × 10^6^	(R)FFAFGR(V)		

## Supplementary Materials

The following are available online. **Table S1.** Soy, pea, milk and beetroot specific proteins detected in powdered mixes of additives; **Table S2.** Peptides specific to cow milk and milk of other mammals; **Table S3.** Soy unique peptides obtained from less abundant proteins and detected in all mixes and burgers; **Figure S1.** Total ion chromatograms of the mixes of functional preparations obtained from in-solution tryptic digests: (A) mix M1 with powdered onion as main differentiating component; (B) mix M2 with milk proteins as main differentiating component; (C) mix M3 with pea proteins as main differentiating component. All three mixes contained also soy proteins and powdered beetroot juice; **Figure S2.** Total ion chromatograms of the cooked burgers manufactured with the addition of mixes of functional preparations: (A) burger B1 containing mix M1 with powdered onion as main differentiating component; (B) burger B2 containing mix M2 with milk proteins as main differentiating component; (C) burger B3 containing mix M3 with pea proteins as main differentiating component; **Figure S3.** Mass spectrum of the soy glycinin G1 unique peptide VFDGELQEGR (2+) obtained from cooked burger (sample B5); **Figure S4.** Mass spectrum of the pea provicilin unique peptide VLLEQQEQEPQHR (3+) obtained from functional additive preparation (mix M3); **Figure S5.** Mass spectrum of the beetroot elongation factor 2 unique peptide EGALAEENMR (2+) obtained from functional additive preparation (mix M1); **Figure S6.** Mass spectrum of the beetroot elongation factor 2 unique peptide FFAFGR (2+) obtained from functional additive preparation (mix M1).
