# Supplementary material for: LC–Q–TOF–MS/MS Identification of Specific Non-Meat Proteins and Peptides in Beef Burgers
Source: Molecules. 2018 Dec 21;24(1):18. doi: 10.3390/molecules24010018 (PMC6337087; doi:10.3390/molecules24010018)
Supplement: Supplementary file 1 [file molecules-24-00018-s001.pdf]

molecules

MDPI

Supplementary Materials

LC–Q–TOF–MS/MS Identification of Specific Non-meat Proteins and Peptides in Beef Burgers

Beata Mikołajczak [1]*, Emilia Fornal [2] and Magdalena Montowska [1]

[1] Department of Meat Technology, Poznan University of Life Sciences, Wojska Polskiego 31, Poznań, 60-624, Poland; beata.mikolajczak@up.poznan.pl; magdalena.montowska@up.poznan.pl
[2] Department of Pathophysiology, Medical University of Lublin, Jaczewskiego 8b, Lublin, 20-090, Poland; emilia.fornal@umlub.pl
* Correspondence: beata.mikolajczak@up.poznan.pl; Tel.: +48-618-487-505

Table S1. Soy, pea, milk and beetroot specific proteins detected in powdered mixes of additives.

Identified protein	Accession no.	Mass (Da)	Matched peptides [1]			Sequence coverage [%] [2]		
			M1	M2	M3	M1	M2	M3
**Soy proteins**								
Seed biotinylated protein	Q39846	67933.9	20	18	17	48.2	40.2	39.1
Seed maturation protein PM36	Q9SWB6	26329.6	8	6	7	51.9	29.6	47.1
P24 oleosin isoform A	P29530	23501.2	6	6	6	26.1	26.1	26.1
Stress-induced protein SAM22	P26987	16771.5	3	3	2	23.4	23.4	13.9
18 kDa seed maturation protein	Q01417	17606.0	4	3	4	26.5	21.9	26.5
Proteinase inhibitor C-II	P01063	9999.9	3	3	3	36.1	36.1	36.1
Proteinase inhibitor D-II	P01064	10323.4	3	3	3	37.3	36.1	37.3
Hydrophobic seed protein	P24337	8815.1	3	2	-	37.5	26.2	-
**Pea proteins**								
Provicilin (Fragment)	P02854	46441.1	-	-	4	-	-	11.9
Vicilin	P13918	70577.1	-	-	3	-	-	7.1
Legumin A2	P15838	59668.1	-	-	3	-	-	16
**Milk proteins**								
β -Lactoglobulin	P02754	20282.3	-	13	6	-	81.4	49.4
α-S1-Casein	P02662	24585.5	-	7	-	-	50.9	-
β -Casein	P02666	25163.9	-	5	-	-	43.7	-
Lactotransferrin	P24627	80051.8	-	6	-	-	9.1	-
Butyrophilin A1	P18892	59960.3	-	6	-	-	18	-
α -Lactalbumin	P00711	16702.7	-	6	1	-	66.9	9.8
Lactadherin	Q95114	48551.1	-	2	-	-	5.3	-
Kappa-casein	P02668	21383.1	-	2	-	-	14.7	-
Milk glycoprotein PP3	P80195	17208.3	-	3	-	-	18.9	-
**Beetroot proteins**								
Elongation factor 2	O23755	94767.3	5	2	7	6.8	3.9	10.9

[1] Number of matched peptides in the database search; [2] Percentage of coverage of the entire amino acid sequence.

Table S2. Peptides specific to cow milk and milk of other mammals.

Parent ion m/z	Mr (exp)	Exp z [1]	Peptide score [2]	Total intensity range	Peptide marker	Protein	Protein score [3]
419.2434	837.4764	2	13.84	$2.65{\cdot}10^7$-$2.83{\cdot}10^7$	(K)ALPMHIR(L)	β-Lactoglobulin	245.95
458.7437	916.4734	2	14.85	$3.42{\cdot}10^7$-$3.62{\cdot}10^7$	(K)IDALNENK(V)		
452.2876	903.5662	2	16.20	$1.80{\cdot}10^7$-$3.70{\cdot}10^7$	(K)TKIPAVFK(I)		
903.1334	2707.3760	2	22.99	$2.56{\cdot}10^6$-$4.00{\cdot}10^8$	(K)VAGTWYSLAMAASDISLLDAQSAPLR(V)		
533.2955	1065.5827	2	16.82	$1.02{\cdot}10^8$-$1.11{\cdot}10^8$	(K)VLVLDTDYK(K)		
771.7598	2313.2588	3	22.75	$4.77{\cdot}10^6$-8.1210^8	(R)VYVEELKPTPEGDLEILLQK(W)		
561.2376	1121.4680	2	11.72	$6.21{\cdot}10^6$-$1.10{\cdot}10^7$	(K)WENGECAQK(K)		
940.0952	2818.2667	3	23.60	$5.43{\cdot}10^7$-$5.99{\cdot}10^7$	(K)YLLFCMENSAEPEQSLACQCLVR(T)		
416.1958	831.3843	2	13.88	$2.45{\cdot}10^6$-$5.09{\cdot}10^6$	(K)EDVPSER(Y)	α-S1-Casein	106.74
634.3572	1267.7045	2	20.13	$2.34{\cdot}10^7$-$4.27{\cdot}10^7$	(R)YLGYLEQLLR(L)		
684.3518	1367.6954	2	10.47	$6.40{\cdot}10^6$-$6.89{\cdot}10^5$	(K)ALNEINQFYQK(F)	α-S2-Casein	53.05
903.8094	2709.4075	3	12.93	$2.58{\cdot}10^6$-$2.89{\cdot}10^6$	(K)FPQYLQYLYQGPIVLNPWDQVK(R)		
415.7296	830.4519	2	14.76	$2.18{\cdot}10^6$-$4.14{\cdot}10^6$	(K)AVPYPQR(D)	β-Casein	55.59
729.3955	2186.1678	3	21.07	$1.48{\cdot}10^7$-$1.66{\cdot}10^7$	(R)DMPIQAFLLYQEPVLGPVR(G)		
727.1165	2905.4367	4	9.92	$1.22{\cdot}10^6$-3.5610^6	(R)ATLHAVDVTLDPDTAHPHLFLYEDSK(S)	Butyrophilin A1	80.89
1002.4135	2003.8182	2	21.48	$3.15{\cdot}10^7$-$3.62{\cdot}10^7$	(K)DDQNPHSSNICNISCDK(F)	α-Lactalbumin	120.03
850.3852	1699.7554	2	26.11	$9.70{\cdot}10^5$-$1.02{\cdot}10^8$	(K)FLDDDLTDDIMCVK(K)		
546.2657	1091.5190	2	19.79	$5.01{\cdot}10^7$-$5.41{\cdot}10^7$	(K)LDQWLCEK(L)		
600.8302	1200.6524	2	18.98	$2.88{\cdot}10^7$-$3.23{\cdot}10^7$	(K)VGINYWLAHK(A)		
626.3592	1251.7096	2	18.97	$1.38{\cdot}10^6$-$3.05{\cdot}10^6$	(K)YIPIQYVLSR(Y)	Kappa-casein	34.99

[1] Parent ion charge state; [2] Spectrum Mill peptide score at FDR of 1%; [3] Spectrum Mill protein score at FDR of 1%.

Table S3. Soy unique peptides obtained from less abundant proteins and detected in all mixes and burgers.

Parent ion m/z	Mr (exp)	Exp z [1]	Peptide score [2]	Total intensity range	Peptide marker	Protein	Protein score [3]
1042.4858	2083.9614	2	22.63	$3.60\cdot10^6$-$1.61\cdot10^8$	(K)FNECQLNNLNALEPDHR(V)	Glycinin	352.21
926.4301	1851.8508	2	21.48	$9.31\cdot10^5$-$1.73\cdot10^8$	(K)HFLAQSFNTNEDTAEK(L)		
1289.6354	5155.5072	4	21.19	$3.15\cdot10^6$-$6.50\cdot10^7$	(R)HFNEGDVLVIPLGVPYWTYNTGDEPVVAISPL DTSNFNNQLDQNPR(V)		
781.0857	2341.2373	3	17.06	$6.51\cdot10^5$-$7.35\cdot10^7$	(R)NGSHLPSYLPYPQMIIVVQGK(G)		
663.8233	1326.6397	2	17.18	$7.14\cdot10^5$-$2.27\cdot10^7$	(R)SQQQLQDSHQK(I)		
910.4396	2729.2988	3	20.73	$7.75\cdot10^6$-$2.82\cdot10^8$	(R)VFYLAGNPDIEHPETMQQQQQQK(S)		
573.2833	1145.5586	2	9.22	$8.38\cdot10^5$-$3.46\cdot10^7$	(K)YQGNSGPLVNP(-)		
509.6322	1526.8788	3	16.72	$7.77\cdot10^6$-$2.52\cdot10^8$	(K)QIVTVEGGLSVISPK(W)	Glycinin/G4	352.21/554.89
839.4053	1677.8013	2	21.74	$2.69\cdot10^5$-$5.60\cdot10^7$	(R)VVNCQGNAVFDGELR(R)		
699.9201	1398.8315	2	20.19	$5.29\cdot10^6$-$1.66\cdot10^8$	(R)ISTLNSLTLPALR(Q)		
785.4238	1569.8384	2	25.67	$5.69\cdot10^6$-$1.15\cdot10^8$	(R)AIPSEVLAHSYNLR(Q)	Glycinin G4	554.89
753.1508	3009.5720	4	18.88	$5.24\cdot10^6$-$6.09\cdot10^8$	(R)GQLLVVPQNFVVAEQAGEQGFEYIVFK(T)		
932.4483	1863.8872	2	21.19	$3.93\cdot10^6$-$1.97\cdot10^8$	(K)HFLAQSFNTNEDIAEK(L)		
1025.4930	2049.9771	2	20.74	$1.56\cdot10^6$-$1.01\cdot10^8$	(K)LNECQLNNLNALEPDHR(V)		
437.2653	873.5226	2	16.27	$3.35\cdot10^6$-$1.17\cdot10^8$	(R)MIIIAQGK(G)		
773.3956	2318.1676	2	26.56	$1.33\cdot10^6$-$5.30\cdot10^7$	(K)NGIYSPHWNLNANSVIYVTR(G)		
793.9334	1586.8577	2	18.71	$2.76\cdot10^6$-$8.23\cdot10^7$	(R)QFQLSAQYVVLYK(N)		
764.0137	2290.0218	3	19.20	$1.21\cdot10^6$-$1.80\cdot10^8$	(K)QGQHQQEEEEEGGSVLSGFSK(H)		
718.0294	2152.0669	3	22.84	$4.71\cdot10^6$-$2.46\cdot10^8$	(R)VESEGGLIQTWNSQHPELK(C)		
919.1084	2755.3032	3	18.69	$1.65\cdot10^6$-$5.09\cdot10^8$	(R)VFYLAGNPDIEYPETMQQQQQQK(S)		
1057.9988	2114.9890	2	23.01	$3.37\cdot10^6$-$1.53\cdot10^8$	(K)YEGNWGPLVNPESQQGSPR(V)		
704.4454	1407.8821	2	20.23	$1.87\cdot10^5$-$3.51\cdot10^7$	(R)DVISTIIPLPVIK(E)	Seed lipoxygenase-1	801.43

952.1921	2854.5561	3	25.44	$1.86\cdot10^{6}-6.95\cdot10^{7}$	(K)LPTLISLSVIEILSTHASDEVYLGQR(D)		
869.0148	1737.0231	2	23.02	$1.37\cdot10^{6}-7.74\cdot10^{7}$	(K)LPTEVISTIMPLPVVK(E)	Seed lipoxygenase-2	773.88
712.7190	2136.1376	3	23.04	$9.54\cdot10^{5}-1.24\cdot10^{8}$	(R)IFFANQTYLPSETPAPLVK(Y)	Seed lipoxygenase-3	826.23
782.7554	2346.2452	3	22.37	$1.23\cdot10^{7}-7.73\cdot10^{7}$	(K)SVSQNVLPLLQSAFDLNFTPR(E)		
637.8341	1274.6587	2	16.83	$2.56\cdot10^{6}-7.05\cdot10^{7}$	(K)DNIVSSLDNVAK(E)	Sucrose-binding protein	477.81
801.0866	2401.2398	3	21.67	$1.26\cdot10^{7}-2.56\cdot10^{8}$	(R)DPESVLSAFSWNVLQAALQTPK(G)		
708.3540	1415.6994	2	20.80	$5.84\cdot10^{5}-5.80\cdot10^{7}$	(K)ESFFFPFELPR(E)		
807.4400	2420.3006	3	19.40	$3.30\cdot10^{6}-4.97\cdot10^{7}$	(K)ITLEPGDMIHIPAGTPLYIVNR(D)		
544.2434	1630.7126	3	24.06	$1.10\cdot10^{6}-2.01\cdot10^{7}$	(K)CGDIGISIDHDDGTR(R)	Trypsin inhibitor A	298.05
530.2848	1059.5615	2	19.01	$1.92\cdot10^{6}-1.01\cdot10^{8}$	(R)CPLTVVQSR(N)		
1102.5553	3305.6473	3	22.06	$9.08\cdot10^{5}-1.77\cdot10^{8}$	(K)FDSFAVIMLCVGIPTEWSVVEDLPEGPAVK(I)		
725.3382	1449.6679	2	22.96	$9.36\cdot10^{5}-4.45\cdot10^{7}$	(K)LVFCPQQAEDDK(C)		
676.3181	1351.6277	2	18.89	$7.98\cdot10^{5}-3.19\cdot10^{7}$	(R)DTVDGWFNIER(V)	Kunitz trypsin inhibitor KTI1	192.16
510.2539	1019.5004	2	14.23	$1.75\cdot10^{6}-9.19\cdot10^{6}$	(K)GGGIEVDSTGK(E)		
722.3864	1443.7631	2	20.83	$1.00\cdot10^{6}-2.38\cdot10^{7}$	(R)ALYSTPIHIWDK(E)	Lectin	203.01
879.9568	1758.9055	2	16.46	$1.48\cdot10^{5}-3.05\cdot10^{7}$	(K)QPNMILQGDAIVTSSGK(L)		
652.3601	1303.7104	2	22.66	$5.85\cdot10^{6}-7.90\cdot10^{7}$	(R)TSNILSDVVDLK(T)		
575.2818	1149.5535	2	14.91	$4.14\cdot10^{5}-1.74\cdot10^{7}$	(K)TTSWDLANNK(V)		
799.4432	2396.3072	3	20.28	$4.31\cdot10^{5}-6.11\cdot10^{7}$	(K)VLITYDASTSLLVASLVYPSQR(T)		
610.8104	1220.6126	2	18.17	$4.54\cdot10^{6}-6.95\cdot10^{7}$	(K)ELINLATMCR(F)	2S albumin	132.36
693.8564	1386.7046	2	19.11	$2.33\cdot10^{6}-5.00\cdot10^{7}$	(K)QLQGVNLTPCEK(H)		
622.7649	1244.5225	2	16.76	$7.43\cdot10^{4}-2.71\cdot10^{7}$	(K)WQHQQDSCR(K)		
529.7855	1058.5629	2	18.57	$2.28\cdot10^{6}-8.00\cdot10^{7}$	(R)YEAGVVPPAR(F)	P24 oleosin isoform B	115.11
573.7807	1146.5538	2	22.92	$2.27\cdot10^{5}-8.57\cdot10^{6}$	(K)APSSIYHSER(G)		

645.2703	1933.7922	3	21.39	$1.84{\cdot}10^{6}$-$7.21{\cdot}10^{7}$	(K)EQYSCDHPPASWDWR(K)	P34 probable thiol protease	107.53
736.3485	2207.0260	3	22.32	$2.06{\cdot}10^{6}$-$1.17{\cdot}10^{8}$	(R)NTGNLLGVCGMNYFASYPTK(E)		
1454.0452	4360.1164	3	22.57	$3.96{\cdot}10^{6}$-$5.59{\cdot}10^{8}$	(K)VTIDGYETLIMSDESTESETEQAFLSAILEQPISVSIDAK(D)		
928.4970	2783.4700	3	23.97	$1.04{\cdot}10^{7}$-$4.95{\cdot}10^{8}$	(R)NTQGVAGLGHAPISLPNQLASHFGLQR(Q)	Basic 7S globulin	281.52
1070.2098	3208.6062	3	19.41	$2.62{\cdot}10^{5}$-$5.02{\cdot}10^{7}$	(R)QFQNQDIFHDLAFTPLTITLQGEYNVR(V)		
721.8737	1442.7387	2	23.44	$1.92{\cdot}10^{5}$-$1.47{\cdot}10^{8}$	(R)VGFSTSSLHSHGVK(C)	Basic 7S globulin	281.52
1129.8029	4516.1779	4	19.38	$6.21{\cdot}10^{5}$-$5.92{\cdot}10^{7}$	(K)GALIFGDAPNNMQQFHNQDIFHDLAFTPLTVTPQGEYNVR(V)	Basic 7S globulin2	224.55
943.2428	3769.9344	4	18.92	$1.33{\cdot}10^{7}$-$2.77{\cdot}10^{8}$	(R)NIQGVAGLGHAPISLPNQLASHFGLQHQFTTCLSR(Y)		

[1] Parent ion charge state; [2] Spectrum Mill peptide score at FDR of 1%; [3] Spectrum Mill protein score at FDR of 1%.

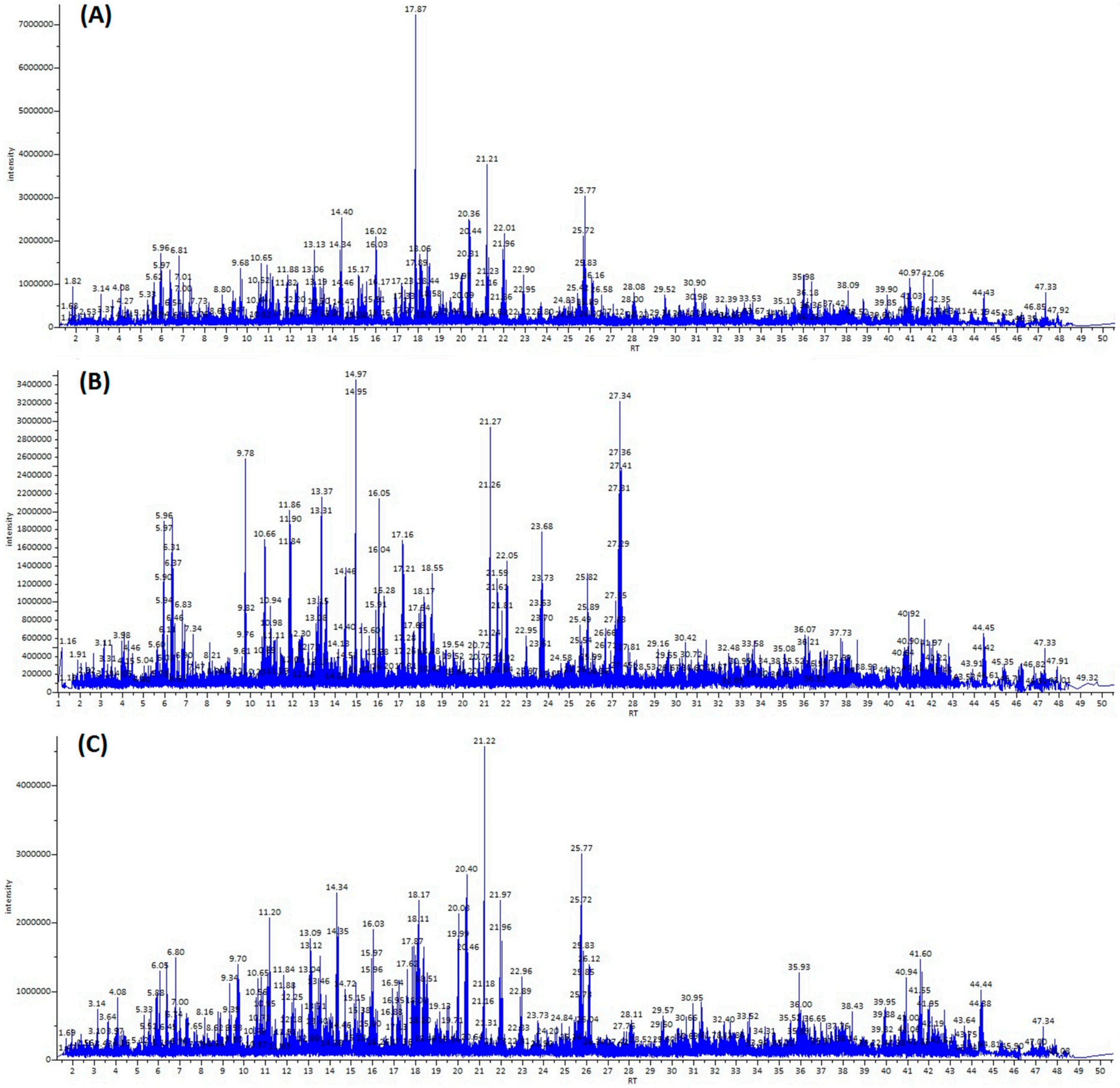

Figure S1. Total ion chromatograms of the mixes of functional preparations obtained from in-solution tryptic digests: (A) mix M1 with powdered onion as main differentiating component; (B) mix M2 with milk proteins as main differentiating component; (C) mix M3 with pea proteins as main differentiating component. All three mixes contained also soy proteins and powdered beetroot juice.

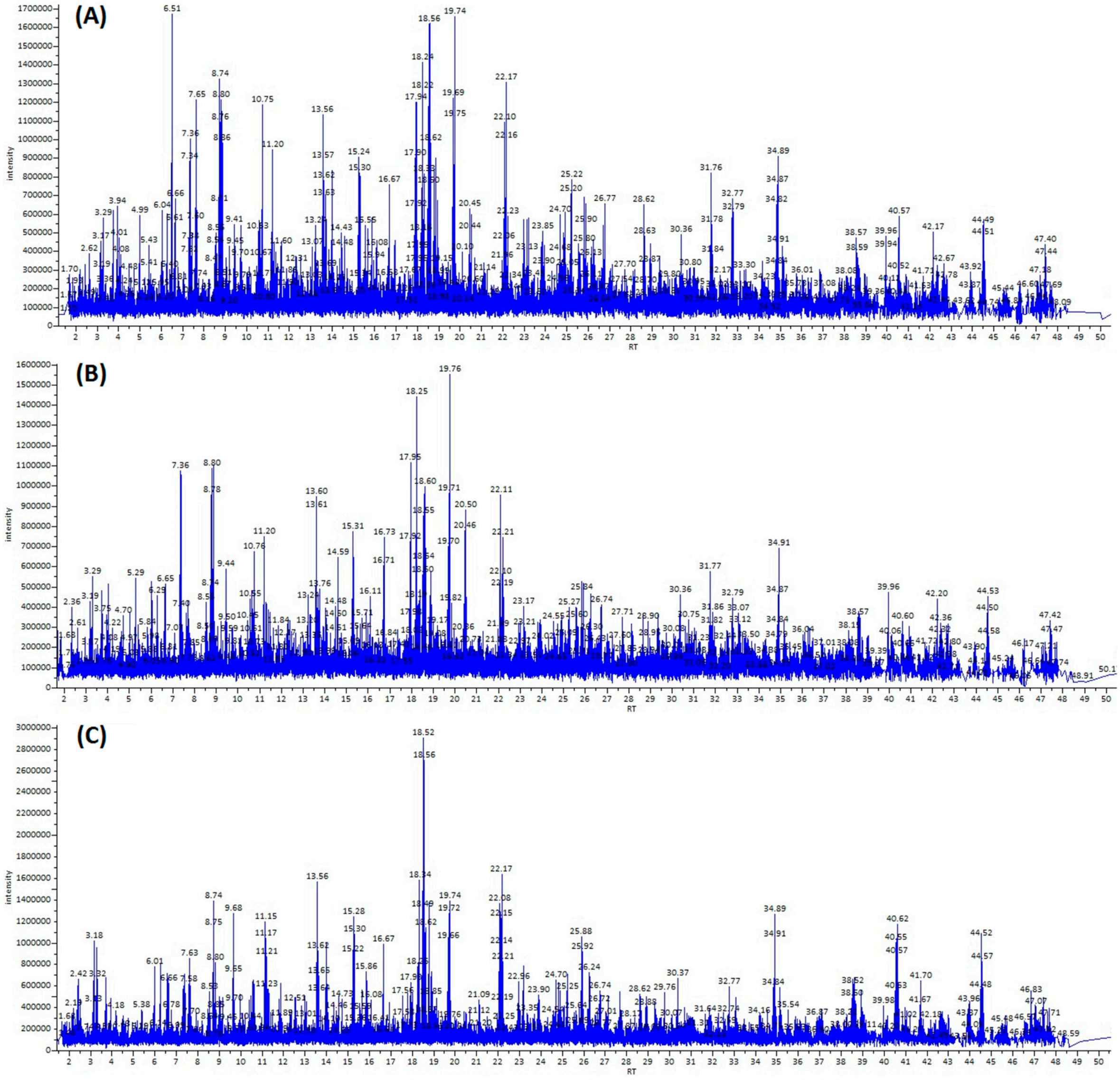

Figure S2. Total ion chromatograms of the cooked burgers manufactured with the addition of mixes of functional preparations: (A) burger B1 containing mix M1 with powdered onion as main differentiating component; (B) burger B2 containing mix M2 with milk proteins as main differentiating component; (C) burger B3 containing mix M3 with pea proteins as main differentiating component.

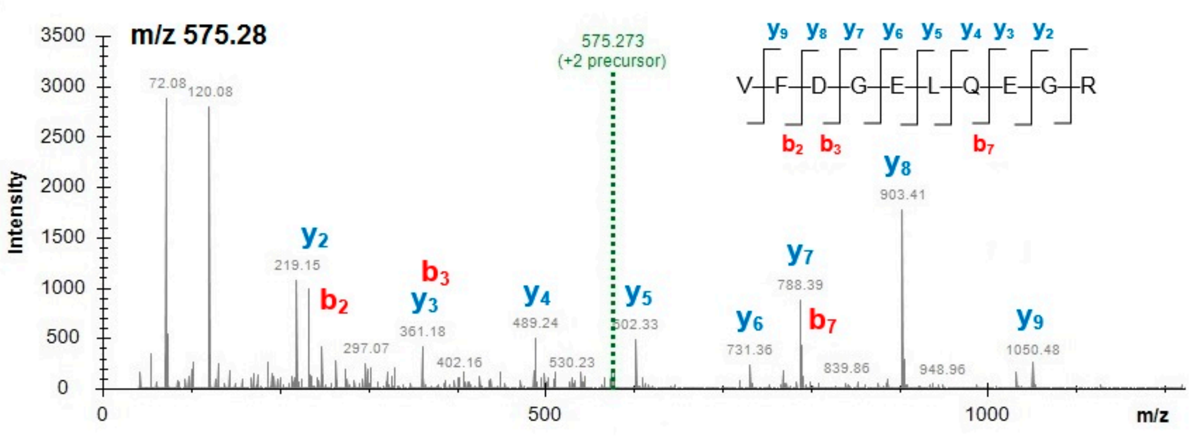

Figure S3. Mass spectrum of the soy glycinin G1 unique peptide VFDGELQEGR (2+) obtained from cooked burger (sample B5).

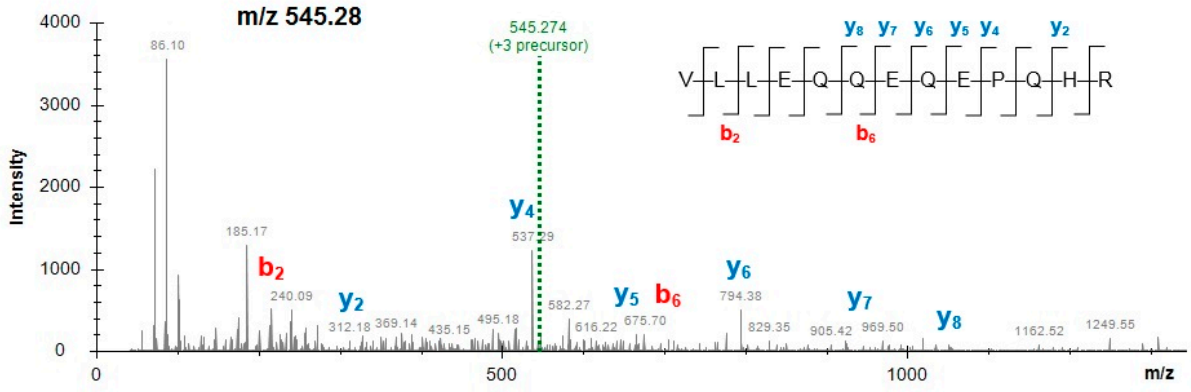

Figure S4. Mass spectrum of the pea provicilin unique peptide VLLEQQEQEPQHR (3+) obtained from functional additive preparation (mix M3).

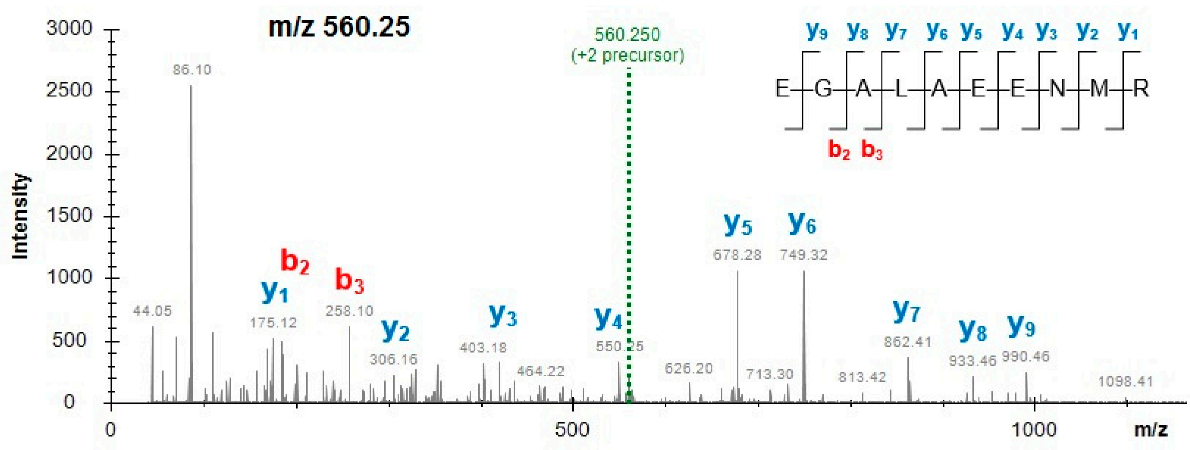

Figure S5. Mass spectrum of the beetroot elongation factor 2 unique peptide EGALAEENMR (2+) obtained from functional additive preparation (mix M1).

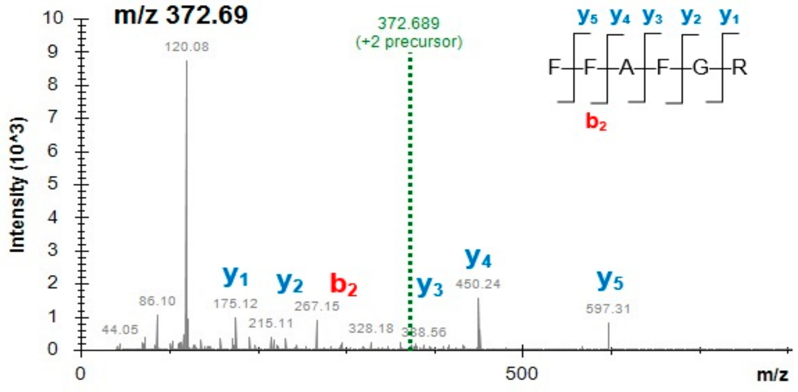

Figure S6. Mass spectrum of the beetroot elongation factor 2 unique peptide FFAFGR (2+) obtained from functional additive preparation (mix M1).
